# Voluntary Medical Male Circumcision: Logistics, Commodities, and Waste Management Requirements for Scale-Up of Services

**DOI:** 10.1371/journal.pmed.1001128

**Published:** 2011-11-29

**Authors:** Dianna Edgil, Petra Stankard, Steven Forsythe, Dino Rech, Kristin Chrouser, Tigistu Adamu, Sameer Sakallah, Anne Goldzier Thomas, Jennifer Albertini, David Stanton, Kim Eva Dickson, Emmanuel Njeuhmeli

**Affiliations:** 1 United States Agency for International Development, Washington, District of Columbia, United States of America; 2 Gillings School of Global Public Health, University of North Carolina, Chapel Hill, North Carolina, United States of America; 3 Futures Institute, Glastonbury, Connecticut, United States of America; 4 Centre for HIV and AIDS Prevention Studies, Johannesburg, South Africa; 5 Jhpiego, Baltimore, Maryland, United States of America; 6 Supply Chain Management Systems, Arlington, Virginia, United States of America; 7 Department of Defense HIV/AIDS Prevention Program, Naval Health Research Center, San Diego, California, United States of America; 8 United States Agency for International Development, Mbabane, Swaziland; 9 World Health Organization, Geneva, Switzerland; Centers for Disease Control and Prevention, United States of America

## Abstract

**Background:**

The global HIV prevention community is implementing voluntary medical male circumcision (VMMC) programs across eastern and southern Africa, with a goal of reaching 80% coverage in adult males by 2015. Successful implementation will depend on the accessibility of commodities essential for VMMC programming and the appropriate allocation of resources to support the VMMC supply chain. For this, the United States President’s Emergency Plan for AIDS Relief, in collaboration with the World Health Organization and the Joint United Nations Programme on HIV/AIDS, has developed a standard list of commodities for VMMC programs.

**Methods and Findings:**

This list of commodities was used to inform program planning for a 1-y program to circumcise 152,000 adult men in Swaziland. During this process, additional key commodities were identified, expanding the standard list to include commodities for waste management, HIV counseling and testing, and the treatment of sexually transmitted infections.

The approximate costs for the procurement of commodities, management of a supply chain, and waste disposal, were determined for the VMMC program in Swaziland using current market prices of goods and services.

Previous costing studies of VMMC programs did not capture supply chain costs, nor the full range of commodities needed for VMMC program implementation or waste management. Our calculations indicate that depending upon the volume of services provided, supply chain and waste management, including commodities and associated labor, contribute between US$58.92 and US$73.57 to the cost of performing one adult male circumcision in Swaziland.

**Conclusions:**

Experience with the VMMC program in Swaziland indicates that supply chain and waste management add approximately US$60 per circumcision, nearly doubling the total per procedure cost estimated previously; these additional costs are used to inform the estimate of per procedure costs modeled by Njeuhmeli et al. in “Voluntary Medical Male Circumcision: Modeling the Impact and Cost of Expanding Male Circumcision for HIV Prevention in Eastern and Southern Africa.” Program planners and policy makers should consider the significant contribution of supply chain and waste management to VMMC program costs as they determine future resource needs for VMMC programs.

*Please see later in the article for the Editors' Summary*

## Abstract 2

Dianna Edgil and colleagues evaluate the supply chain and waste management costs needed to deliver mobile medical male circumcision services to 152,000 men in Swaziland, finding that per-procedure costs almost double when these factors are taken into account.

## Editors’ Summary

**Background:**

About 33 million people (including 22.5 million in sub-Saharan Africa) are currently infected with HIV, the virus that causes AIDS. Although antiretroviral drugs keep HIV in check, there is no cure for HIV/AIDS. Consequently, prevention of HIV transmission is extremely important. Because HIV is usually spread through unprotected sex with an infected partner, individuals can reduce their risk of becoming infected with HIV by abstaining from sex, by having only one or a few partners, and by always using male or female condoms. In addition, trials in sub-Saharan Africa have shown that male circumcision—the removal of the foreskin, the loose fold of skin that covers the head of the penis—reduces the risk of HIV infection in men by 60%. In 2007, the World Health Organization (WHO) and the Joint United Nations Programme on HIV/AIDS (UNAIDS) recommended that voluntary medical male circumcision (VMMC) should be part of HIV prevention programs in regions with a generalized HIV epidemic and a low level of male circumcision. Together with the United States President’s Emergency Plan for AIDS Relief (PEPFAR), WHO, and UNAIDS also prioritized 14 countries in eastern and southern Africa for VMMC program scale-up. Mathematical models suggest that, if 80% VMMC coverage is reached by 2015 (which will entail performing 20.33 million circumcisions between 2011 and 2015) and sustained thereafter, VMMC programs in these priority countries will avert more than 4 million HIV infections among adults between 2009 and 2025.

**Why Was This Study Done?:**

Successful VMMC scale-up will depend on the commodities that are essential for VMMC services being accessible and on the appropriate allocation of resources to support VMMC programs (which, in addition to circumcision, include HIV testing and counseling, sexually transmitted infection screening and treatment, condom provision and promotion, and counseling on risk reduction and safer sex). To help program planners and policy makers, costing studies have been undertaken in several African countries. These studies considered the costs of a standard list of commodities prepared by PEPFAR, WHO, and UNAIDS and estimated that, on average, one male circumcision costs about US$53. However, these studies did not include the costs of the supply chain, waste management, HIV counseling and testing, treatment of sexually transmitted infections, or the temporary infrastructure needed to deliver mobile VMMC services. Here, the researchers estimate these hitherto ignored costs for the Accelerated Saturation Initiative (ASI; *Soka Uncobe* [Circumcise and Conquer] in SiSwati), a one-year program to circumcise 152,000 men in Swaziland.

**What Did the Researchers Do and Find?:**

The researchers used current market prices of goods and services to calculate the fixed and variable costs of various aspects of the VMMC commodity supply chain such as procurement, international freight, in-country distribution to service delivery sites, and warehousing, and of various aspects of waste management, such as the transportation of waste to incinerators and the maintenance of incinerators. They also estimated the staffing costs of supply chain and waste management services. From these component costs, the researchers estimate that, overall, the costs of supply chain and waste management, including procurement of commodities and associated labor, add US$58.92 if 152,000 men are circumcised and US$73.57 if 75,000 men are circumcised to the previously estimated cost of performing one adult male circumcision through the Swaziland ASI VMMC program.

**What Do These Findings Mean?:**

This study suggests that, for the Swaziland ASI VMMC program, procurement, supply chain, and waste management costs nearly double the previously estimated cost per VMMC procedure. That is, the supply chain and waste management costs for this program are nearly as high as the costs of the equipment and staff needed to do the circumcisions. Because these costs were not taken into account during the planning stages of Swaziland’s ASI VMMC program, the initial needs assessment for this program underestimated the actual costs by about US$8 million. Although the magnitude of this underestimate cannot be generalized to other settings, this analysis emphasizes the importance of considering the contribution of supply chain and waste management to costs when determining the future resource needs of VMMC programs. Moreover, it provides a framework to help program planners and policy makers estimate the costs involved in the scale-up of VMMC programs in other priority countries.

**Additional Information:**

Please access these websites via the online version of this summary at http://dx.doi.org/10.1371/journal.pmed.1001128.

- This study is part of a PLoS collection of articles on VMMC (http://www.ploscollections.org/VMMC2011) and is further discussed in a *PLoS Medicine* Review Article by Hankins et al. (http://dx.doi.org/10.1371/journal.pmed.1001127).
- Information is available from WHO, UNAIDS, and PEPFAR on all aspects of HIV/AIDS
- NAM/aidsmap provides basic information about HIV/AIDS, summaries of recent research findings on HIV care and treatment, and information on male circumcision for the prevention of HIV transmission
- Information is available from Avert, an international AIDS charity on many aspects of HIV/AIDS, including information on all aspects of HIV prevention, and on HIV/AIDS in Africa and in Swaziland (in English and Spanish)
- The Clearinghouse on Male Circumcision, a resource provided by WHO, UNAIDS, and other international bodies, provides information and tools for VMMC policy development and program implementation
- Personal stories about living with HIV/AIDS are available through Avert, through NAM/aidsmap, and through the charity website Healthtalkonline

## Introduction

Based on evidence from randomized controlled clinical trials conducted in Africa indicating that voluntary medical male circumcision (VMMC) significantly reduces male participants’ risk of acquiring HIV infection, the global HIV prevention community has supported the scale-up of VMMC programs in 14 countries [1]–[3]. Evidence suggests that reaching a goal of 80% VMMC coverage in 5 y and sustaining it thereafter would avert more than 3.6 million adult HIV infections in the next 15 y and benefit as many as 20.3 million adult HIV-negative men for HIV prevention purposes [4]. Reaching this population with comprehensive VMMC services will have substantial implications for the coordination of human resources [5], commodities, and infrastructure.

Supply chain considerations and commodity costs carry significant financial implications for all public health programs, but these costs are particularly relevant for highly technical programs planned on the scale anticipated for VMMC services. The minimum package of services that should compose all VMMC for HIV prevention programs—as delineated by the World Health Organization (WHO)—encompasses an array of activities beyond circumcision surgery, including (1) HIV testing and counseling, (2) sexually transmitted infection (STI) screening and treatment, (3) condom provision and promotion, and (4) counseling on risk reduction and safer sex [6]. Thus, program needs are broad, and resources must support all major components of a logistics system involving human capital and/or material inputs, procurement of commodities, freight management, warehousing, in-country distribution, and waste management.

As a result, costing studies have been undertaken in Namibia [7], Kenya [8], South Africa [9], Uganda [10], Zambia [11], and Zimbabwe [12] to help governments fully appreciate the initial financial investments required to launch VMMC initiatives.

To date, these studies have estimated the cost of a single male circumcision to be, on average, approximately US$53, ranging from US$19 in Uganda [10] to US$73 in Zambia [11]. However, until this study, no quantification of VMMC program costs, to our knowledge, has included the full range of supply chain and commodity considerations. For example, the supply chain costing data included by Njeuhmeli et al. [4] uses costing data collected in Zimbabwe in 2009 that estimated the per circumcision supply chain costs at between US$59.71 and US$71.59 ([12]; Supply Chain Management Systems–Zimbabwe, personal communication). While the Zimbabwe exercise went further than its predecessors in looking at the cost of procurement and distribution of commodities, it failed to account for all necessary supply chain costs associated with VMMC programs, including warehousing and waste management. Gaps in data present difficulties for both policy makers and implementers as they plan for the rapid scale-up of adult VMMC.

The Partnership for Supply Chain Management (PFSCM), supported by the United States President’s Emergency Plan for AIDS Relief (PEPFAR), began assisting countries that were scaling up VMMC programs to develop, strengthen, and manage reliable, cost-effective, and sustainable supply chains as early as 2009. Further, in an attempt to harness economies of scale and streamline logistical issues, PEPFAR hosted an expert consultation in 2009 on VMMC supply chain issues [13].

As a result of that meeting, a standard list of VMMC commodities was put forward by international stakeholders. The bundling of commodities into surgical instrument kits was recommended (depending upon surgical method and disposability of items), as well as standardized equipment (for infection prevention, emergency care, and general surgical theater equipment) [13]. The meeting did not address supplies for HIV testing, STI screening or treatment, or equipment for waste management. In 2010, the WHO’s release of “Considerations for Implementing Models for Optimizing the Volume and Efficiency for Male Circumcision Services” (MOVE) clarified the need for standardized commodities and bundled supplies and equipment to achieve efficiency [14].

Given the limitations of previous costing exercises for VMMC programs, the purpose of this paper is to present, to our knowledge for the first time, the full supply chain costs associated with the scale-up of a VMMC program in one southern African country, Swaziland. This information will also improve the standard list of commodities outlined by PEPFAR and WHO by adding commodities for waste management, counseling and testing, and temporary infrastructure for VMMC programming.

To inform this analysis, we collected costing information for supply chain and waste management activities and commodities associated with the planned implementation of Swaziland’s 1-y VMMC scale-up campaign—known as the ASI [15]. This information was used to calculate the total cost per VMMC for the Swazi program with either a planned target of 152,000 VMMCs or a reduced target of 75,000 VMMCs. In our analysis of costs required for the ASI, we outline key commodity and supply chain inputs and offer future considerations that may serve to inform VMMC program planning and policy development in coming years.

### Swaziland VMMC Program Service Provision Model

The Swazi campaign goal is to increase VMMC coverage for HIV prevention purposes from 8% to 80% among 15- to 49-y-old males by the end of 2011 to avert nearly 100,000 HIV infections; reaching this target equates to circumcising approximately 152,000 men [15]. In line with WHO MOVE recommendations for efficient service delivery, the Swazi service provision strategy includes the use of prepackaged VMMC kits with disposable surgical instruments [14]. Optimum implementation of the service delivery model chosen by Swaziland enables a service delivery team—composed of a surgeon assisted by five nurses and four counselors—to deliver services to an average of 40 clients per day, with a maximum throughput of 80 clients per day.

Consistent with MOVE recommendations and to meet the target of 152,000 VMMCs, it was determined that services would be delivered by a varying number of teams per month (to account for demand seasonality and scale-up and scale-down periods), with up to 35 teams operating simultaneously at 27 sites during peak months in 2011 [14]. Seven of these sites (comprising 14 teams) are considered fixed and are integrated into existing health facilities. The remaining sites, all but one of which comprise one team each (the exception has two teams), are considered mobile, and are contained within tented facilities, which, for the purpose of meeting unexpected demand, can be moved to a different location.

At the midpoint of the VMMC campaign in Swaziland, the rate of scale-up is less than would be expected to allow the program to reach its target of 152,000 VMMCs. To reflect a lower rate of service delivery, with a target of reaching 75,000 men over 1 y, the number of operational sites was reduced to 20, with a single team operating at each site.

The PFSCM provides technical and programmatic support on procurement, logistics, and waste management to the Government of the Kingdom of Swaziland in its effort to implement the ASI program. In support of the Swaziland campaign, and with input from other stakeholders, PFSCM developed a full list of commodities for the ASI campaign. This included the four modules already developed by PEPFAR and WHO, as well as commodities modules for (1) HIV counseling and testing and STI treatment and (2) temporary infrastructure necessary for the delivery of mobile VMMC services (full contents of modules and line item costs are listed in Table S1). Furthermore, the module on infection prevention was expanded to include all commodities necessary for waste management for VMMC programs. These modules were added to the four modules already developed by PEPFAR [13] and WHO [14].

In addition to assisting with the development of the complete list of commodities, PFSCM was asked to provide technical assistance on the development of a supply chain to deliver necessary commodities for the ASI campaign. The structure of the supply chain for this program included quantification, forecasting, procurement, freight forwarding, customs negotiation, warehousing, implementation of a supply chain management information system for stock keeping and ordering, distribution of commodities when needed to VMMC sites, and monitoring of supplies at the site level. Three staff at the organization’s headquarters in Arlington, Virginia, performed the quantification, forecasting, and procurement activities. The warehousing and distribution of commodities to VMMC sites was contracted through the local private sector market. With this contract, PFSCM retained the use of sufficient warehouse space and racking for all commodities for the ASI program over an 18-mo period, allowing for program scale-up and scale-down. As well, the local contractor provided trucks and drivers to accommodate both high- and low-demand scenarios for the in-country distribution of supplies. PFSCM employed three full-time staff in Swaziland to oversee management of the warehouse, the management information system, the contractor, and monitoring of the sites.

PFSCM also provided technical assistance to support the Government of the Kingdom of Swaziland Ministry of Health in increasing the capacity and sustainability of the current waste disposal system while integrating programmatic VMMC waste disposal needs. At the national level, this included the revision of national health care waste management guidelines and policies and supporting personnel with training, supervision, and tools for record keeping and documentation. At the facility level, waste management costs include transporting medical waste from the VMMC sites to the regional hospitals for final disposal; regularly monitoring and evaluating the plan to ensure that practices are maintained properly to minimize risk, damage, and disease transmission; procuring required waste disposal commodities (including incinerators) to ensure proper waste storage, handling, and disposal; and developing training curricula for facility staff responsible for waste management [16].

## Methods

### Unit Cost Analysis for the Calculation of Commodities, Logistics, and Waste Management Costs in Swaziland

In addition to the programmatic costs associated with the operation of the supply chain, we assessed the fixed and variable cost of all quantified commodities, organized into six modules: (1) VMMC kit and consumables, (2) infection prevention and waste management, (3) equipment for male circumcision sites, (4) emergency medical supplies, (5) HIV counseling and testing and other STI management, and (6) temporary infrastructure. (Full contents of modules and line item costs are listed in Table S1.)

The following sections detail the assumptions we made in calculating the unit cost for the ASI service delivery model.

### Service Utilization

In calculating the unit cost of a single VMMC, start-up or fixed costs, including equipment costs, are divided by the number of circumcisions achieved. This process assumes that the utilization of the VMMC facilities is at a sufficient volume of clients to allow for 152,000 circumcisions to be performed over the course of 12 mo. Per VMMC costs were also re-analyzed using a reduced target of 75,000 VMMCs to reflect the current rate of approximately 6,500 VMMCs per month.

### Variable Commodity Costs

We calculated consumables costs based on high-volume price quotes secured by PFSCM. The calculated cost of freight for all commodities is 6% of the total cost. In addition to the cost of consumables, we calculated a 5% surcharge for procurement services provided by the Arlington-based staff of PFSCM. This cost covers the effort required to source suppliers, compile tender documents, negotiate with suppliers, and assess the quality of proposals and actual products.

### Fixed Commodity Costs

We derived unit costs for fixed-cost items for all sites from price quotes obtained by PFSCM based on the commodities outlined in Table 1. These items include those on the standard list of essential equipment as well as infrastructure commodities needed to establish a mobile site.

**Table 1:** Supply chain costs.

Category	Sub-Category	Target of 152,000 VMMCs	Target of 75,000 VMMCs
Personnel and in-country operation costs	Staff	$246,720.00	$246,720.00
	Office operation costs	$37,100.00	$37,100.00
Supply chain costs	Warehouse and cross docking in Matsapa	$2,400.00	$2,400.00
	Domestic distribution to 27 sites	$160,245.00	$160,245.00
	24-h security coverage at warehouses	$26,190.00	$26,190.00
	In-bound cartage JNB–MTS^a^ plus customs clearance	$45,045.00	$45,045.00
	International freight	$431,792.22	$257,474.41
	Commodity insurance	$11,939.05	$7,119.17
	Fuel costs for site operations	$105,840.00	$105,840.00
	Fuel costs for incineration	$109,500.00	$109,500.00
	Distribution of waste from five regions/40 sites to region waste center	$160,245.00	$160,245.00
	Incinerator maintenance	$39,250.00	$39,250.00
Fees	Non-facility-based technical assistance overhead	$21,488.00	$21,488.00
	Non-facility, non-labor overhead	$2,095.00	$2,095.00
	Procurement fee	$359,826.85	$214,562.01

All costs given in US dollars.

a Johannesburg Grand Central Airport, South Africa, to Manzini-Matsapha International Airport, Swaziland.

### Supply Chain Costs

Next are supply chain management costs, including warehousing, distribution, and other logistical services required to deliver products to sites, as well as staffing costs. For the program, PFSCM hired local staff for in-country logistics and waste management positions. The salary for the logistics advisor position was calculated based on local salaries. Personnel costs for a locally hired logistics advisor were at rates exceeding local government rates, but 50% lower than expatriate rates, to account for the need to ensure the services of skilled staff.

The calculated cost of non-facility-based overheads is 12% of in-country operating costs, to account for technical assistance and travel on the part of the PFSCM Project Management Office. The cost of in-country supply chain and logistics staff was divided by the total number of targeted male circumcisions (152,000) or the revised target (75,000) for a single year of the VMMC scale-up program.

In addition to the costs identified above, we added 2% of the total direct operating costs to cover overhead costs that were neither facility- nor labor-based. For example, the 2% overhead would cover communication costs and other program-management-related expenses.

### Waste Management Operational Costs

Waste management costs are both fixed and variable. PFSCM performed an assessment of the national waste management system prior to developing the waste management plan for the Swazi VMMC program. Based on the volume and weight of the commodities needed for each VMMC using fully disposable kits, PFSCM assessed the needs of the waste disposal system in Swaziland, using the combined weight of the consumables required for an individual surgery to produce an estimate of approximately 0.5 kg of biologically contaminated waste to be generated per procedure. This level was more than the national waste disposal system could process.

In this analysis, staffing for waste management included the provision of technical assistance by two PFSCM headquarters staff and the training and support of two local staff (a driver and a waste handler) for transportation of medical waste. As well, cost sharing of incinerator operation and staff time at regional hospitals was included in the final cost.

## Results

Using the expanded list of WHO-recommended VMMC commodities, we estimated the commodity procurement, supply chain, and waste management costs associated with providing VMMC services to 152,000 adult men in 1 y in Swaziland to be US$8,956,213.46. This is the equivalent of US$58.92 per VMMC. For a reduced target of 75,000 VMMCs in 1 y, the total cost is calculated as US$5,518,393.50, the equivalent of US$73.57 per VMMC.

### Module 1: VMMC Kit and Consumables

Module 1 contains the ethylene-oxide-sterilized, fully disposable kit for performing the forceps-guided method of VMMC and supplementary consumable medical supplies. These commodities are required for the performance of each surgery, representing a variable cost of US$25.17 per circumcision. In the Swaziland context with a target of 152,000 VMMCs, Module 1 costs contribute a total of US$3,839,144.08 to the commodity and supply chain costs of the program. For a target of 75,000 VMMCs, Module 1 contributes US$1,902,572.04 to the total cost of the Swazi VMMC program, representing a per VMMC cost of US$25.18. 

Aligned with prior PEPFAR and WHO guidance, the VMMC kit comprises a consumables pack and an instrument set [14]. The consumables pack contains a standardized set of disposable items necessary for the performance of one male circumcision. This includes gauze, a scalpel, syringes, gloves, aprons, sutures, needles, surgical tape, alcohol swabs, and an O drape. The instrument set contains all necessary surgical instruments for the performance of one circumcision. 

Items within the kit are packed in the order in which they will be required for the surgical procedure. Before being placed in the kit, consumable items and any necessary instruments are removed from the manufacturer’s external packaging. This reduces the total VMMC procedure time by removing the need to unwrap various commodities. Removal from the original packaging does destroy the items’ sterility, necessitating that the entire kit be sterilized using ethylene oxide gas before use. This service is provided by the kit packager and included in the total cost of the kit. The market price for an ethylene-oxide-sterilized VMMC kit with a standard consumables pack and disposable forceps-guided instrument set is currently US$17. 

Module 1 also includes bulk consumables that supplement those found in the VMMC kit. These include pharmaceuticals necessary for the procedure (paracetamol, lignocaine, and betadine) but that were removed from the kit itself due to expiration concerns and importation restrictions. Additional sutures, gloves, and gauze also are included, in case those in the kit are not adequate. The cost of these consumables is estimated to be US$8.26 per VMMC.

### Module 2: Infection Prevention and Waste Management

The second module contains standard infection prevention items as well as those required for waste management. Previously, based on WHO recommendations, this module contained only infection prevention commodities. However, given the importance of effective waste management and its potential impact on the broader health system, this estimate expands the module to include waste management commodities.

Most infection prevention commodities are consumable and represent variable costs, depending on the number of circumcisions performed. Waste management commodities represent largely fixed costs that require one-time procurement at the start of program operations. In Swaziland, for a target of 152,000 VMMCs, these commodities total US$1,427,853.90 and contribute US$9.39 to the cost of each VMMC. To reach a target of 75,000 VMMCs, the total cost of commodities for Module 2 is US$819,862.91, contributing US$10.93 to the cost of each VMMC. Of these costs, US$211,872 is fixed.

Infection prevention commodities, while critical to program implementation, are largely low cost and include items such as surgical masks and caps, protective eyewear, surgical scrub solution and alcohol hand wash, buckets for instrument cleaning, and bleach.

In contrast, waste management commodity costs can be significant. Items such as sharps bins and biohazard bags are used to collect three types of medical waste associated with a VMMC program—sharps waste (two needles, one surgical blade, and one to two sutures), human tissue (foreskin), and general medical waste (used gauze, gloves, etc.). This waste is transported to an incinerator, where it is turned into an ash by-product. For Swaziland, the ASI program will require the provision of upgrades to the current waste disposal facilities and the procurement of two additional incinerators, as well as the generators and fuel necessary to operate these incinerators. This increases the cost of Module 2 by US$105,470 (US$0.69 per VMMC).

Metal waste, generated by the use of either disposable or reusable instruments, cannot be incinerated and must be buried or recycled. These disposal methods do not include additional commodity costs, but do require that the instruments be disinfected prior to disposal. In the case of the ASI program, disinfected metal waste will be donated to a metal foundry in Swaziland. Transportation costs to the foundry are included in the cost of metal waste disposal.

### Module 3: Equipment for Male Circumcision Sites

The third module includes equipment necessary for the set-up of one operating theater with four beds and one recovery area. This includes such items as an operating table, lamp, instrument tray, intravenous stand, diathermy machine, and recovery chair. Small equipment items, such as a stethoscope and glucometer, are also included. These items represent fixed costs, requiring procurement only once during program implementation. Glucometer strips may be considered an exception, as they will require replacement over the lifetime of a program.

For a program reaching a target of 152,000 VMMCs, with service provision at 27 sites and 35 teams, the total cost of this module is US$469,202.33, or US$3.08 per VMMC, US$2,100 of which is variable costs. For a reduced target of 75,000 VMMCs achieved through service provision at 20 sites with 20 teams, this module contributes US$267,953.96 (US$1,050 variable costs) to the total cost of the Swazi VMMC program, or US$3.57 per VMMC.

### Module 4: Emergency Medical Supplies

The final WHO-recommended module, emergency medical supplies, includes items necessary in the case of a medical emergency. These include jump bags with necessary medical equipment (such as glucometers, oxygen cylinders, and sphygmomanometers), emergency pharmaceuticals (atropine, adrenaline, dextrose, and sodium chloride), and an emergency trolley to hold these items. With the exception of the pharmaceuticals, these costs are fixed. Items in the jump bag may require occasional replacement, but this cost will be minimal and dependent on the number of adverse events in a program. These replacement costs are not included in this calculation.

In Swaziland, for a program targeting 152,000 VMMCs through service provision at 35 sites, the total cost of this module is US$50,022.92, or US$0.33 per VMMC. Of these costs, US$49,630 is fixed. For a VMMC program reaching 75,000 men through service provision at 20 sites, the total cost of this module is US$28,556.46, of which US$28,360 is fixed. This module contributes US$0.38 to the cost of each VMMC.

### Module 5: HIV Counseling and Testing and Other STI Management

This module includes HIV test kits, lancets, and pipettes, as well as pharmaceuticals for the treatment of STIs. These costs are variable, and the quantity required will be determined by the rate of consumption. For example, HIV tests should be recommended to all VMMC clients, making this cost dependent on expected demand. Confirmatory test kits should be ordered based on the HIV prevalence in the target population. Similarly, pharmaceutical costs for STI management will depend on the prevalence of various STIs in the population.

In Swaziland, the total cost of this module is US$486,833.70, or US$3.20 per VMMC to reach a target of 152,000 VMMCs. To reach a target of 75,000 VMMCs, this module costs US$243,416.85, or US$3.25 per VMMC.

### Module 6: Temporary Infrastructure

Finally, two key commodities are included as a separate module and represent costs associated with the mobile delivery of VMMC services in Swaziland. The first comprises mobile medical (marquees) and industrial tents where VMMC mobile services will operate. The second is generators, which will be used to power diathermy machines and other electrical equipment at mobile sites. Also included are costs associated with fuel to operate the generators.

We believe that these are important costs to assess, given that many VMMC programs are considering mobile delivery of services. While the delivery method remains nearly identical in these mobile sites, there are key commodity considerations. In Swaziland, where tent structures will be used for 23 out of 27 sites (the remaining sites will use existing structures), these commodities contribute US$921,380 to the total cost of VMMC service delivery. This is the equivalent of US$6.06 per circumcision. Reducing the number of sites providing VMMC services to 20 results in an overall reduction in the total cost of this module to US$835,130, with a per VMMC cost of US$11.14. Despite the reduction of the VMMC target by half, the program still requires the use of 20 sites to facilitate access to services over the entire country. This results in a near doubling of the per VMMC costs in this module despite a reduction in volume.

### Supply Chain Costs

The procurement, freight, and distribution costs associated with these commodities are significant. After estimating costs associated with each step of the supply chain, the total cost of these services amounts to US$1,759,676.52. Costs included in the supply chain calculation are found in Table 1. This is reduced to US$1,435,273.99 with a reduction in target to 75,000 VMMCs. The reduction is determined by the calculated procurement service fee as a percentage of total commodity costs.

Logistics costs include both international freight and in-country distribution to 27 service delivery sites. We also include commodity insurance and clearance costs based on current market rates, costs associated with warehousing commodities in Swaziland, and the costs of moving commodities from a central warehouse to individual surgical sites. Finally, we include the cost of transporting waste to incineration sites and performing maintenance on incinerators.

Last, we assess additional costs associated with in-country operation costs, particularly personnel. We include a waste management supervisor, as well as resources for monitoring and evaluation and technical assistance, given the embryonic state of waste management services in Swaziland. We include five additional staffing positions for other supply chain and waste management services, as well as expenses associated with an office for these staff. Staffing and other operation costs amount to US$283,820.

### Total Cost of Commodities and Supply Chain Management

Based on the above modules, we estimate the total cost of commodities, supply chain, and waste management for the Swaziland VMMC program reaching a target of 152,000 men to be US$8,956,213.46. Approximately US$2,609,202.66 of these costs is fixed, while the remaining US$6,334,010.80 is variable, based on the number of VMMCs performed, the number of medical staff, and the number of surgical sites. Fixed and variable costs are outlined by module in Table 2.

**Table 2:** Fixed and variable costs, by module.

Module	Target of 152,000 VMMCs		Target of 75,000 VMMCs	
	Fixed	Variable	Fixed	Variable
VMMC kits and consumables	None	$3,839,144.08	None	$1,902,572.04
Infection prevention and waste management	$211,871.93	$1,215,981.98	$211,871.93	$607,990.99
Equipment for male circumcision sites	$469,202.33	$2,100.00	$266,903.96	$1,050
Emergency medical supplies	$49,630.00	$392.92	$28,360.00	$196.46
Counseling and testing and other STI management	$1,000.00	$485,833.70	$500	$243,416.85
Temporary infrastructure	$921,380.00	None	$835,130.00	None

All costs given in US dollars.

The total cost of commodities, supply chain, and waste management is US$58.92 per male circumcision.

For a reduced target of 75,000 VMMCs, the total cost is calculated as US$5,518,393.50, which equals a per VMMC cost of US$73.57. Fixed and variable costs for this reduced target are also included in Table 2.

## Discussion

This report represents the first attempt, to our knowledge, to quantify fully the supply chain and waste management costs associated with VMMC programming. Previous VMMC program costing studies generally have been performed at the point of service provision and thus have failed to capture supply chain and waste management costs. However, given the significant contribution to total program costs made by commodities, it is critical that these costs be considered during implementation planning to provide an accurate indication of the resources required.

For example, based on previous costing studies, which indicated an average cost of US$50 per VMMC, the initial resource needs assessment for the ASI program underestimated the actual costs by approximately US$8 million. For program planners, an underestimate of this magnitude would gravely impact successful program implementation. Moreover, given Joint United Nations Programme on HIV/AIDS (UNAIDS) estimates of a US$10,000,000,000 funding gap for HIV programs globally [17], accurate program costs are critical to determining the most efficient and effective use of resources to impact the global epidemic.

This paper provides a quantification that represents the best international guidance on commodity procurement. For this reason, the analysis takes into account commodities not previously considered, including HIV counseling and testing, STI treatment, temporary infrastructure, and waste management; the last is particularly important, given the historical lack of attention to this area and its potential impact on strengthening the overall health system [7]–[12]. So, too, is temporary infrastructure given the increasing understanding by program planners and policy makers that outreach services are an integral component of all VMMC programs.

The data presented here represent rapid scale-up of VMMC in a low-resource setting with minimal supply chain systems. These assumptions impact the calculations provided and limit their generalization to other country settings. Various factors need to be considered before this calculation is used to inform costing estimations in other settings.

First, better-resourced countries may incur lower program costs because of existing infrastructure and supply chain functions (e.g., warehousing, waste management). For example, according to the estimates reported here, commodities make up a significant proportion of the total unit cost (US$47.34–US$49.50). Consumables contribute more than two-thirds of the supply chain costs for VMMC programs. These costs may be reduced below the estimates presented here by using existing infrastructure (i.e., existing operating tables) and strategically placing pooled orders, which take advantage of economies of scale and allow for the negotiation of better pricing on bulk commodities. Similar use of existing infrastructure could reduce costs associated with the procurement of temporary infrastructure, which adds at least US$6.06, and as much as US$11.14, to the cost of each surgery (e.g., tents, generators). Additionally, the use of in-country as opposed to expatriate staff can result in considerable savings. In the case of Swaziland, staffing costs can be reduced by at least 50% through hiring skilled local staff.

The implementation timeline may also alter costs. The costs associated with the ASI program in Swaziland assume the generation of demand for 152,000 or 75,000 VMMCs in 12 mo; a lengthier timeline needed to meet these targets would increase costs for rentals, warehousing and distribution contracting, staffing, and some formerly bulk-procured commodities. Countries planning to perform a larger number of VMMCs over a longer period of time will see some cost savings as certain flat-rate costs are distributed across more surgeries (e.g., surgical beds, diathermy machines).

Program planners will also need to consider the fact that the number of VMMC sites will impact fixed costs and change resource needs. These costs will increase with the number of sites selected by program planners. The selection of the appropriate number of sites for a particular campaign would be determined by such factors as volume over a given time frame, geography, size, difficulty of transport, and population density. However, given that many VMMC programs are expected to move quickly to full scale and reach target saturation within 2–3 y of initiation, many of the costs of increased targets simply would be multiplied by the number of teams and/or sites at a commensurate increased cost. Alternatively, the volume through an individual site (assuming a maximum capacity of 80 VMMCs per team per day based on the MOVE model) is associated with variable costs, which would increase as volume increases, while the fixed costs associated with each site would decrease as volume increases.

The largest single cost in consumables is that of the disposable kit, at US$1,275,000–US$2,584,000. Prices for forceps-guided disposable kits were negotiated by PFSCM for the aggregate group of instruments (rather than by line item), including packaging and sterilization. We did this to take advantage of economies of scale, not to be directive regarding VMMC method. The disposable kit seems relatively expensive but does help to improve efficiency and avoids the need for sterilization equipment and staff training. In fact, through PFSCM, the pooled procurement of VMMC kits already has resulted in a reduction in market price from US$23 to US$17 per kit. Further reductions in prices are expected as additional VMMC kit vendors enter the market. As of March 2011, 6 mo after the procurement of VMMC kits for Swaziland, that cost had already dropped to US$15 per kit.

While the Swazi ASI chose to use the forceps-guided VMMC technique, some programs have chosen to use other methods of VMMC (i.e., sleeve resection or dorsal slit). These techniques require different and/or additional instruments, require more time per procedure, and are less commonly used in VMMC programs. Program planners choosing to use one of these methods can expect an increase in commodity costs for the prepackaged kit, which is currently priced at US$19 per kit. As well, increasing the time required per surgery results in a decrease in the volume of clients serviced per team at the site level, resulting in increased fixed costs.

As discussed, some programs may opt for reusable rather than disposable instruments. However, reusable instrument “lifetimes” vary significantly because of variations in instrument quality and differences in cleaning and maintenance practices. In addition, there are costs for reusable instrument use (in equipment purchase, maintenance, training, human resource time expenditures, cleaning solutions, and instrument turnaround time), so one cannot assume that reusable instruments are more cost-effective in every context. Programs with pre-existing autoclave facilities and staff that can be expanded (or fully utilized, if not currently at capacity) are the most likely to derive cost savings from using reusable instruments. Some programs might choose a combination of reusable instruments at sites with autoclaves and disposable kits at less well-equipped mobile and outreach sites [14]. Such variable patterns of program design have significant cost impacts that are difficult to quantify and cannot be reflected fully in this report.

In addition, this report assumes the use of a diathermy machine for cauterization, which changes costs associated with instruments by decreasing the number of sutures required. Programs considering the use of diathermy machines should consider costs associated with additional staff training required to operate these machines safely, as such costs are not reflected in this paper. Only programs with a reliable electricity supply, stable voltage, and demand for high-volume VMMC services are likely to derive significant benefit from the investment in diathermy machines.

Finally, in Swaziland, the costs of waste disposal were based on the use of incineration for biological waste disposal. For other countries, costs will be impacted for programs choosing alternative disposal methods. As mentioned previously, countries can adapt cost estimates to reflect the reality of their infrastructure (which might vary from site to site). As in the case of Swaziland, improvements made to the health care waste management system for the disposal of VMMC programmatic waste can greatly improve waste management abilities throughout the health care system.

### Lessons Learned

VMMC programs implemented at the scale and pace required to stem HIV transmission and save lives as quickly as possible are somewhat at odds with the global health community’s focus on strengthening health systems. The WHO MOVE recommendations attempt to mitigate demands on the health care system by task shifting, creating efficient patient flows, and simplifying the complex list of commodities needed for a successful program. Still, much like vaccination campaigns, the implementation of VMMC programs often involves the creation of a parallel supply chain for VMMC commodities outside of the national essential medicines supply chain; this parallel supply chain is generally managed by the implementing partner providing VMMC services. These commodities are varied (requiring the management of over 50 different consumable products alone), demand large well-ordered storage facilities, and must be distributed to a range of dedicated and mobile clinics with seasonal operating schedules and locations.

In the case of Swaziland, and given the lack of precedent in the literature, supply chain and logistics needs to support the ambitious program implementation plan were vastly underestimated and under-budgeted. Commodities included in the initial action plan were limited to VMMC kits and HIV rapid test kits; the plan for the management of the supply chain in Swaziland was limited to procurement, delivery to a small warehouse for temporary cross docking, and near immediate distribution to VMMC service provision sites. The staffing plan included only a logistics advisor and an administrative assistant.

The exercise documented here helped program planners address this challenge, vastly improving estimation and budgeting for the Swaziland program. Several key lessons learned from this process will be useful to other VMMC programs in Africa to ensure efficient resource allocations.

First, proper assessment of programmatic needs can prevent programs from being under-resourced. For example, because over two-thirds of the total supply chain budget is devoted to the procurement of nearly 100 different commodities, the act of quantifying program needs allowed for a more realistic budget to be created (an increase from US$2.5 million to US$8.9 million). Without this, the VMMC program in Swaziland would have been under-resourced, stalling before a single procedure was performed.

Second, assessment of needs and cost estimation also ensure that existing resources are properly allocated. In Swaziland, the waste management system assessment conducted prior to launch of the program was critical to ensuring appropriate resource allocation. The assessment was conducted out of a recognition that because most African waste management programs are unable to meet current waste disposal demands [18], the effort to reach national targets through VMMC programs will quickly overwhelm national waste disposal systems. As a result of the assessment, the costs estimated for the management of VMMC-program-generated waste were significantly reduced, and the estimated number of incinerators needed decreased from six to two. This reduction in anticipated waste management costs allowed for reallocation of funding resources to other programmatic priorities.

Finally, despite extensive needs assessment and cost estimation, program implementation can still result in unexpected supply chain costs associated with the implementation of a VMMC program. In the Swaziland context, funding delays, the reality of lead times associated with any procurement process, and slow community engagement led to a late start to program activities (start delayed from January to April). This meant that at the midpoint of the campaign, communication efforts had generated less demand for service than anticipated. This has significantly impacted supply chain and logistics needs on the ground.

Commodities were initially ordered to support a program meeting a target of 152,000 VMMCs over a 1-y period. With a lower than expected demand for services, the supply chain for these commodities has required a great deal of change. From a cross-docking operation that quickly moved commodities to the field for immediate consumption, at the country level, the Swazi VMMC supply chain has evolved into a complicated operation involving warehouse management, distribution, oversight of commodities consumption and waste disposal practices at the service delivery site, forecasting of needs, and reverse logistics of program-generated waste. Ultimately, from an initial plan of hiring two staff, five were hired to manage the supply chain, in addition to the expansion of the scopes of contracts for warehousing and distribution of supplies.

In addition, for VMMC kits (US$17 per kit) procured in January 2011 for a 1-y program, a 2-y shelf life was rapidly being spent. At the current rate of service provision, the program was projected to reach 75,000 VMMCs at the end of the 1-y period. Given this forecast, it was decided that of 100,000 kits currently in country, 40,000 should be sold internationally. This required packaging, transportation, and customs brokerage, but allowed a savings of nearly US$1 million in programmatic costs that might have been wasted had the commodities expired in the warehouse.

This implementation experience speaks to an important lesson learned regarding the necessity of staging the importation of commodities into a country based on real consumption data. A balance exists between staging orders and a desire to save money by pooling small orders into a much larger one; for untested VMMC programs, this desire should be tempered by the uncertainty of demand in country.

### Limitations

While we include line item costs in this paper for one VMMC scale-up program, commodity and service costs can vary significantly, depending on location, order volume, and other market forces. The widely variable setting in which VMMC scale-up is being undertaken has such a significant impact on line item values as to make them almost impossible to estimate for broad applicability. However, this paper provides a framework for supply chain and waste management cost considerations. In addition, varying quality of such items can impact cost (and usable lifetime) significantly, and it is difficult to differentiate specific commodities in terms of quality (aside from field testing, for which only anecdotal data are available).

In addition, although certain commodities are listed as fixed costs (operating table, trolleys, instrument stands), there may be additional costs not included here, such as the cost of maintenance and repair. Such costs are difficult to estimate and will depend on such factors as the volume of clients, climate, quality of routine maintenance, and total time used. Due to the significant variability of such costs from country to country, programs will need to make procurement decisions based on their particular settings and take into account the additional costs associated with such repairs in their specific context. Additionally, costs for resupply of emergency drugs in case of adverse events are not included.

Another limitation to this paper is that the data for commodities are derived from current market prices for quantities calculated at an estimated rate of consumption, rather than expenditure reporting. It is quite likely that prices of certain commodities will be impacted by supply/demand issues both locally and internationally. Moreover, if there is a wide variation in the specific commodities ordered by program planners, the potential economies of scale can be lost or diluted.

This variation in commodity costs is a particular limitation for the cost estimates provided for the performance of 75,000 VMMCs. Because PFSCM negotiated prices based on an initial volume of 152,000 VMMCs, the line item costs used for this estimate reflect this higher bulk pricing. It is possible that if 75,000 VMMCs had been the program target at the outset, rather than a function of decreased demand, commodity costs would have been higher because of lower volumes during pricing negotiations. This may have resulted in a further increase in the per VMMC price.

Also, this paper does not take into account the impact of scale-up strategies (such as numbers of sites and VMMC volumes per site over time) or the need for and cost of different commodities over time. However, where possible, we have outlined fixed and variable costs associated with VMMC scale-up. These calculations should be assessed carefully based on local context to avoid over- or underestimation of costs. For example, if a site is not operating at full capacity, high fixed costs will increase the per procedure cost at that facility.

The implementation time period (scale-up, scale-down) is not included as a variable in the calculations in this paper. The time over which services are delivered can be leveraged to maximize cost efficiencies. For example, as VMMC coverage goals are met, the demand drops and low-volume sites can be closed. The remaining usable commodities at these sites (beds, lights, instruments) can be used to replace at active sites those commodities that are worn out or in need of repair, thus reducing repair/replacement costs.

Finally, this report estimates costs associated with VMMCs performed according to current WHO guidelines [19]. Research into novel technologies, including circumcision devices and methods of local anesthesia administration, are ongoing throughout Africa, and these technologies will likely be more broadly available within the next 2–3 y. These technologies have the potential to transform the VMMC service provision model and may drive commodity prices lower, resulting in additional cost savings for the intervention. The prospective use of such technologies, the costs of training staff in performing new techniques, and the need to revise VMMC kit composition and other associated commodities should be considered by program planners in the transition between the old and new procedures for future VMMC programs as new information becomes available.

## Conclusion

As VMMC programs mature, further efforts regarding costing should be undertaken to improve on the current estimates of the cost of VMMC. Experience from implementing the ASI VMMC program in Swaziland indicates that supply chain and waste management add an additional approximately US$60 per VMMC, which adds nearly 100% in additional costs per procedure to program costs. This analysis provides a framework to inform program planning with a more accurate representation of the critical supply chain and waste management costs of VMMC services.

## Supporting Information

Table S1 Commodities and price list.
(DOCX)

## Abbreviations

MOVE: the World Health Organization report “Considerations for Implementing Models for Optimizing the Volume and Efficiency for Male Circumcision Services”
PEPFAR: United States President’s Emergency Plan for AIDS Relief
PFSCM: Partnership for Supply Chain Management
STI: sexually transmitted infection
UNAIDS: Joint United Nations Programme on HIV/AIDS
VMMC: voluntary medical male circumcision
WHO: World Health Organization
