# Supplementary material for: Voluntary Medical Male Circumcision: Logistics, Commodities, and Waste Management Requirements for Scale-Up of Services
Source: PLoS Med. 2011 Nov 29;8(11):e1001128. doi: 10.1371/journal.pmed.1001128 (PMC3226460; doi:10.1371/journal.pmed.1001128)
Supplement: Table S1 — Commodities and price list. (DOCX) [file pmed.1001128.s001.docx]

| **Supplemental Table 1: Commodities and Price List** |  |  |  |
| --- | --- | --- | --- |
| **Commodity** | **Fixed/ Variable** | **Quantity** | **Price** |
| **MODULE 1: MMC KIT AND ADDITIONAL CONSUMABLES** | | | |
| *MMC Kit* | V | 152,000 | 2584000.00 |
| Multipurpose Container Tray – Stable recyclable plastic tray to conduct procedure. Minimum 700 micron virgin plastic with 4 compartments |  | 1 | - |
| O-Drape-Disposable, 100*75cm |  | 1 | - |
| Gauze, Plain Swabs – 100X100mm (12-ply) |  | 20 | - |
| Gauze, Petroleum Jelly – 10x10cm (1-ply) |  | 1 | - |
| Syringe, 10ml |  | 1 | - |
| Injection Needles, 21g and 23g |  | 1 of each gauge | - |
| Suture, Braided/absorbable – 3/0 braided synthetic (polyglycolic acid suture), 75cm, on reverse cutting needle, 26mm |  | 2 | - |
| Sterile surgical gloves (sizes 8 and 7.5) |  | 1 of each size | - |
| Apron, Disposable, plastic |  | 2 | - |
| Alcohol Swabs, 1 ¼ x 2 ½, isopropyl alcohol 70% |  | 2 | - |
| Surgical tape micropore 12mm, length 1–3m |  | 1 | - |
| Sterile Prep Gloves, examination glove large |  | 1 | - |
| Combination needle-holder and scissors, total length 12–14cm, working surface 20mm |  | 1 | - |
| Non-toothed plastic forceps, total length 13 cm, working surface 15mm, serrated |  | 1 | - |
| Mosquito clamps, straight, haemostatic, total length 12–14cm, working surface 30mm |  | 1 | - |
| Mosquito clamps, curved, haemostatic, total length 12-14cm, working surface 30mm |  | 1 | - |
| Disposable Scalpel and Handle, retractable and lockable, blade type 23, total length 11cm |  | 1 | - |
| Circumcision Forceps, haemostatic cross-clamp, total length 20cm, working surface 64mm |  | 1 | - |
| *MMC Additional Consumables* |  |  |  |
| Gloves, surgical latex, powdered, sterile, size 6.5 | V | 160,000 pair | 95301.60 |
| Gloves, surgical latex, powdered, sterile, size 7 | V | 240,000 pair | 123099.65 |
| Gloves, surgical latex, powdered, sterile, size 7.5 | V | 240,000 pair | 135954.00 |
| Gloves, surgical latex, powdered, sterile, size 8 | V | 160,000 pair | 88616.40 |
| Examination Glove, 100 per box | V | 45,500 boxes | 368580.33 |
| Syringe, sterile, disposable, 10ml | V | 34,000 | 5830.26 |
| Needles, hypodermic, sterile, 18 G | V | 34,000 | 3610.61 |
| Needles, hypodermic, sterile, 21 G | V | 34,000 | 1407.22 |
| Needles, hypodermic, sterile, 23 | V | 34,000 | 1407.22 |
| Bandage, self-adhesive, elastic, 7.5cm x 4m | V | 32,560 | 36486.67 |
| Gauze, sterile, 100mm x 100mm, 12 ply, 100pcs/box | V | 16,000 | 247232.67 |
| Gauze, paraffin, 10cm x 10cm | V | 128,000 | 72448.00 |
| Swabs, alcohol, 65mm x 35mm, 200 pcs/box | V | 800 | 4438.13 |
| Linen, saver | V |  | 32141.33 |
| Lidocaine HC1%, 20ml, 20 vials per box | V | 950 boxes | 7457.50 |
| Paracetamol 500mg (1,000 tablets/unit) | V | 3,600 units | 17892.00 |
| Povidone Iodine, 10% solution, 200 ml | V | 13,650 | 13240.50 |
| *TOTAL COST* |  |  | *3839144.09* |
| *TOTAL COST PER MMC* |  |  | *25.26* |
|  |  |  |  |
| **MODULE 2: INFECTION PREVENTION AND WASTE MANAGEMENT** | | | |
| Incinerator | F | 2 | 5,000 |
| Ash pit tanks | F | 12 | 20,400 |
| Ash pit safety fencing | F | 6 | 27,000 |
| Lockable trash containers, 90 gallon | F | 210 | 35,070 |
| Lockable trash containers safety fencing | F | 6 | 18,000 |
| Protective eyewear | F | 655 | 1276.01 |
| Sharp boxes, paper, 5 liters | F | 62 | 5844.07 |
| Small medical plastic bin, 15 liters | F | 52 | 979.57 |
| Large medical plastic bin, 50 liters | F | 48 | 2342.12 |
| Buckets for instrument disinfection and soaking, 10 liter bottles | F | 300 | 18831.72 |
| Surgical mask, 1-ply, disposable, 20 pcs/box | V | 2548 boxes | 6806.7 |
| Surgical cap, disposable, 100 pcs/box | V | 510 boxes | 7374.44 |
| Biohazard trash bag, 15 liter, 200 pcs/box | V | 906 boxes | 74322.09 |
| Biohazard trash bag. 50 liter, 100 pcs/box | V | 906 boxes | 71399.19 |
| Instrument brush. 360mm and bristles of 120x50mm | F | 600 | 3338.00 |
| Utility gloves | F | 145 | 30375.33 |
| Surgical scrub for providers Chlorhexidine, 4%, 3.785 liter | V | 2,466 | 111863.82 |
| Alcohol hand washes for providers, contains isopropanol, ethanol, n-propanol or a combination of these ingredients, 3.785 liter | V | 252 | 124781.64 |
| Soap for scrubbing instruments, contains enzymes that dissolve proteinaceous material, 3.785 liter | V | 1628 | 308585.20 |
| Bleach for soaking instruments, 3.5% sodium hypochlorite, 1 gallon | V | 1628 | 51305.74 |
| Pants, moli, large | V | 76,000 | 225558.00 |
| Pants, moli, medium | V | 76,000 | 216318.60 |
| Scrubs suit, medium | F | 720 | 15040.08 |
| Scrubs suit, large | F | 720 | 15261.80 |
| Scrubs suit, extra large | F | 720 | 16451.24 |
| Overshoes, plastic, blue, 100pcs/box | V | 1600 boxes | 14328.56 |
| *MODULE 2 TOTAL COST* |  |  | *1427853.92* |
| *MODULE 2 TOTAL COST PER MMC* |  |  | *9.39* |
|  |  |  |  |
| **MODULE 3: MMC EQUIPMENT** | | | |
| Operating stool, adjustable height | F | 70 | 8,636.59 |
| Operating table, table, examination, folding, 2-section with washable pad, minimum height of 68cm | F | 175 | 60,333.67 |
| Standing lamp, standing one-bulb spotlight/lamp with adjustable arm | F | 140 | 37,526.06 |
| Step ladder, 1 step, anti-slip rubber, chrome plated steel, plastic-covered feet | F | 140 | 8,128.42 |
| Intravenous stand, 2 hooks on 5 castors, adjustable from 115 to 210 cm | F | 35 | 4,630.39 |
| Recovery chair | F | 140 | 149,752 |
| Instrument stand, mayo stand | F | 100 | 16,257.06 |
| Head Torches | F | 70 | 1,607.74 |
| Spray bottles | F | 55 | 240 |
| Diathermy machine, monopolar | F | 70 | 81,130 |
| Diathermy accessories: Plate | F | 100 | 22,200 |
| Diathermy accessories: Pencil | F | 175 | 19,775 |
| Diathermy accessories: Foot pedals | F | 105 | 16,275 |
| Diathermy accessories: Hand piece | F | 35 | 7,665 |
| Diathermy accessories: Neutral electrode hand piece and or plate | F | 175 | 17,850 |
| Diathermy accessory: Electrode blade | F | 800 | 9,600 |
| Glucometer | F | 35 | 1,750 |
| Glucometer strips, 50 strips/box | V | 70 boxes | 2,100 |
| Sphygmomanometer, aneroid, 300 mm Hg, with adult cuff (for arm diameter approximately 9–14 inches) | F | 35 | 245 |
| Stethoscope, binaural, standard, dual head | F | 35 | 245 |
| Laryngoscope, battery-operated, with three blades (#1, #2, #3), either Miller or MacIntosh | F | 35 | 3255 |
| *MODULE 3 TOTAL COST* |  |  | *469,202.33* |
| *MODULE 3 TOTAL COST PER MMC* |  |  | *3.09* |
|  |  |  |  |
| **MODULE 4: MMC EMERGENCY EQUIPMENT** | | | |
| Adrenaline (epinephrine) 1mg/ml, 100 ampules/unit | V | 4 units | 40.00 |
| Atropine Sulphate 1mg/ml, 100 ampules/unit | V | 4 units | 32.20 |
| Dextrose 50%, 50ml, 20 vials/unit | V | 10 units | 160.40 |
| Sodium Chloride 0.9%, 1000ml, with nipple, 12 bottles/unit | V | 16 units | 160.32 |
| *Jump Bag with the contents below:* | F | 70 | 49630.00 |
| Non-rebreather oxygen mask and oxygen tubing |  | - | - |
| Ambu bag |  | - | - |
| Pen torch, battery operated |  | - | - |
| Oropharyngeal airway, transparent, size 3 (96 mm) |  | - | - |
| Oropharyngeal airway, transparent, size 4 (103 mm) |  | - | - |
| Oropharyngeal airway, transparent, size 5 (120 mm) |  | - | - |
| Glucometer |  | - | - |
| Glucometer Strips |  | - | - |
| Sphygmomanometer, aneroid, 300 mm Hg, with adult cuff (for arm diameter approximately 9–14 inches) |  | - | - |
| Stethoscope, binaural, standard, dual head |  | - | - |
| Tourniquet Small elastic, 90 x 5 cm |  | - | - |
| Laryngoscope, battery-operated, with three blades (#1, #2, #3), either Miller or MacIntosh |  | - | - |
| I.V. infusion tubing (connects bottle or bag of fluid with canula in patient’s vein). At least one injection port required through which to give drugs |  | - | - |
| I.V. catheter, 18 G x 1.75 inch (1.3 x 45 mm) with port & wings, sterile, disposable |  | - | - |
| I.V. catheter, 22 G x 1 inch (0.9 x 25 mm) with port & wings, sterile, disposable |  | - | - |
| I.V. catheter, 16 G x 1 inch (0.9 x 25 mm) with port & wings, sterile, disposable |  | - | - |
| Tape, to secure I.V. catheters and ET tubes |  | - | - |
| Pump, aspirating, surgical Pump, aspirating, surgical, portable, foot-operated, capacity up to 600 mm Hg |  | - | - |
| Endotracheal tube, CH 6, 50 cm, disposable, sterile |  | - | - |
| Endotracheal tube, CH 7, 50 cm, disposable, sterile |  | - | - |
| Endotracheal tube, CH 8, 50 cm, disposable, sterile |  | - | - |
| Oxygen cylinder, 10-liter, with regulator |  | - | - |
| Disposable exam gloves medium, box of 100 |  | - | - |
| Alcohol swabs, individually packed. |  | - | - |
| Gauze 4 x 4 inch (or 10 x 10 cm) in packages of 2 |  | - | - |
| Syringe 2 cc |  | - | - |
| Syringe 10 cc |  | - | - |
| Needle 23 gauge |  | - | - |
| Needle 21 gauge |  |  | - |
| *MODULE 4 TOTAL COST* |  |  | *50022.92* |
| *MODULE 4 TOTAL COST PER MMC* |  |  | *0.33* |
|  |  |  |  |
| **MODULE 5: HIV COUNSELING AND TESTING AND SEXUALLY TRANSMITTED INFECTION TREATMENT** | | | |
| Determine , HIV 1/2 test w/buffer, test card-10 cards (10 tests per card) | V | 450 | 36000.00 |
| Buffer, chase (for UNIGOLD), 2.5ml bottle | V | 1,270 | 6350.00 |
| UNIGOLD, HIV 1/2 Test (20 tests- kit contents- 20 testing devices, wash reagent (2 ml), 20 disposable pipettes, 1 package insert) | V | 6,350 | 203200.00 |
| Lancets | V | 78000 | 3900.00 |
| Penis Models (2 circumcised, 2 uncircumcised) | F | 50 of each type | 1000.00 |
| BAGS, for drugs, labeled, 1000 per box | V | 1,820 boxes | 193476.30 |
| Benzyl benzoate 25% application | V | 890 liters | 2500.90 |
| Clotrimazole 1% cream, 1 tube of 20g | V | 7500 tubes | 1725.00 |
| Ceftriaxone 250mg, 10 vials/unit | V | 350 units | 1424.50 |
| Benzathine penicillin 2.4 MIU, powder for injection, 50 vials per unit | V | 100units | 1486.00 |
| Water for Injection 10mlx100 ampules | V | 50 units | 158.00 |
| Aciclovir 200mg, 40x25 blister packets (1,000 tablets) | V | 600 blisters | 26442.00 |
| Chlorphenamine maleate, 4mg tablets (1,000 tablets/unit) | V | 400 units | 840.00 |
| Ciprofloxacin 500 mg tablets (100 tablets/unit) | V | 100 units | 500.00 |
| Doxycycline, 100mg tablets (1000 tablets/unit) | V | 84 units | 1071.00 |
| Erythromycin, 500mg tablets (1000 tablets/unit) | V | 20 units | 1160.00 |
| Metronidazole, 200mg tablets (1000 tablets/unit) | V | 1400 units | 5600.00 |
| *MODULE 5 TOTAL COST* |  |  | *486833.70* |
| *MODULE 5 TOTAL COST PER MMC* |  |  | 3.20 |
|  |  |  |  |
| **MODULE 6: TEMPORARY INFRASTRUCTURE** | | | |
| Lockable Steel Cabinets, 2 door, 1 per site | F | 35 | 26250.00 |
| Generator | F | 35 | 70000.00 |
| Air conditioning units | F | 35 | 52500.00 |
| Refrigerator without freezer | F | 35 | 52500.00 |
| Mobile surgical marquees | F | 23 | 720130.00 |
| *MODULE 6 TOTAL COST* |  |  | *921380.00* |
| *MODULE 6 TOTAL COST PER MMC* |  |  | *6.06* |
|  |  |  |  |
